# Blue-light background impairs visual exogenous attention shift

**DOI:** 10.1038/s41598-022-24862-7

**Published:** 2023-03-07

**Authors:** Chien-Chun Yang, Sei-ichi Tsujimura, Su-Ling Yeh

**Affiliations:** 1 Department of Psychology, National Taiwan University, Taipei, Taiwan; 2 Faculty of Design and Architecture, Nagoya City University, Nagoya, Japan; 3 Graduate Institute of Brain and Mind Sciences, National Taiwan University, Taipei, Taiwan; 4 Neurobiology and Cognitive Science Center, National Taiwan University, Taipei, Taiwan; 5 Center for Artificial Intelligence and Advanced Robotics, National Taiwan University, Taipei, Taiwan

**Keywords:** Psychology, Human behaviour

## Abstract

Previous research into the effects of blue light on visual-spatial attention has yielded mixed results due to a lack of properly controlling critical factors like S-cone stimulation, ipRGCs stimulation, and color. We adopted the clock paradigm and systematically manipulated these factors to see how blue light impacts the speed of exogenous and endogenous attention shifts. Experiments 1 and 2 revealed that, relative to the control light, exposure to the blue-light background decreased the speed of exogenous (but not endogenous) attention shift to external stimuli. To further clarify the contribution(s) of blue-light sensitive photoreceptors (i.e., S-cone and ipRGCs), we used a multi-primary system that could manipulate the stimulation of a single type of photoreceptor without changing the stimulation of other photoreceptors (i.e., the silent substitution method). Experiments 3 and 4 revealed that stimulation of S-cones and ipRGCs did not contribute to the impairment of exogenous attention shift. Our findings suggest that associations with blue colors, such as the concept of blue light hazard, cause exogenous attention shift impairment. Some of the previously documented blue-light effects on cognitive performances need to be reevaluated and reconsidered in light of our findings.

## Introduction

Blue light has several physiological and cognitive effects on humans. Blue light, for example, affects the circadian rhythm at night, delaying sleep onset, suppressing melatonin release, and elevating core body temperature^1,2^. Blue light also impacts cognition: it enhances dynamic vision^3,4^, working memory^5–8^, and alertness^9,10^ while slowing subjective time perception^11^.

### Potential factors of blue-light effects

Most research on how blue light impacts physiology and cognition^1–11^ has focused on the functions of *intrinsically photosensitive retinal ganglion cells*^12–14^ *(ipRGCs)*, which contain the blue-light sensitive (460 ~ 480 nm) photopigment— melanopsin. ipRGCs are involved in cognitive processing since they project to brain areas such as the locus coeruleus (LC) and superior colliculus (SC), which influence cognitive functions like alertness and eye saccades^8,15–17^, respectively.

In addition to ipRGCs, some blue light researchers focus on the functions of another type of retinal cells called *S-cones*, not only due to their blue-light-sensitive (peak at 420 nm) and sluggish properties^18,19^ but also because of their sending inhibitory outputs to ipRGCs^20,21^. The two types of photoreceptors, ipRGCs and S-cones, have opposing effects on physiological responses such as pupillary light responses and melatonin suppression^22,23^.

Beyond the low-level photoreceptors, the *blue colo*r in blue lights may be crucial for cognitive processing^24–27^, although it is frequently neglected. Colors can assist or impede human cognitive performances depending on the learned associations^24,25^. For example, the blue background is associated with openness and calmness, which might help with performances in complex tasks and those requiring creativity^26,28,29^ whereas the red background is connected to danger and avoidance, which could narrow the scope of attention^26,30^ and impair achievement-related performances^31^. Although people usually associate blue color with positive concepts^32^, it is unknown if these associations are passed over to blue light.

Additionally, due to the growing use of electronics, people may attribute retinal damage to blue light due to a greater focus on photochemical eye dangers or the blue light hazard^33^. As people are attempting to avoid blue light worldwide, Google searches for "blue light glasses" have multiplied hundreds of times since 2010s^34^. It is possible that people have developed a fear or aversion of blue light, which then affect their visual-spatial attention.

### Visual-spatial attention

Visual-spatial attention, which can be deployed to retrieve visual information from a certain location either exogenously or endogenously^35,36^, is an essential and pervasive component of perceptual and cognitive processes. Exogenous attention is an involuntary system that corresponds to an automatic orienting response to a location where sudden stimulation has occurred, such as a flashed advertisement shown on the website. In contrast, endogenous attention is the voluntary system that fits our ability to willfully monitor information at a given location, as when students view the teaching materials in the classroom^37^. The temporal natures of these two types of attention shifts reflect the efficiencies of perceptual- and cognitive-driven visual attention processing; it takes about 75–175 ms to shift exogenous attention and 300 ms to shift endogenous attention^38–40^.

Although several studies have researched how blue light affects visual-spatial attention, the results are inconclusive^41–44^. The discrepancy in results could be due to a lack of rigorous light manipulation. More specifically, the light manipulations in those blue light studies included changes in various critical factors, including luminance, color, and photoreceptor stimulation, all of which might have confounded the results. The present study aimed to control and manipulate each factor to investigate how they affect the speed of exogenous and endogenous attention shifts.

### Paradigms for evaluating the speed of exogenous and endogenous attention shift

Several paradigms, including the orienting paradigm^36^, the attention-gating paradigm^45^, and Wundt's clock paradigm^38,46^, were developed to determine the speed of visual-spatial attention. The orienting paradigm requires participants to shift their attention to a target preceded by a spatial cue about where to move their attention. In this paradigm, the response times (RTs) of correct trials were used to infer the speed of visual-spatial attention. The other two paradigms ask participants to shift their attention to a continuously-changing object and report its status at the time of the shift (usually at the cue onset). Specifically, participants need to report the letter in a rapid serial presentation stream in the attention-gating paradigm and the clock time on a running clock in Wundt’s clock paradigm, respectively. Based on the temporal lags between a true and a reported letter in the attention-gating paradigm and clock time in the clock paradigm, these two paradigms calculate the speed of spatial attention.

### Hypotheses and aim

We examined how ipRGCs, S cones, and colors affect the shift speed of visual attention and tested four hypotheses with two contrasting views based on low-level perceptual and high-level cognitive processing (Table 1). In terms of the two competing hypotheses based on low-level perceptual processing, blue light could either speed up attention shift^47,48^ because the ipRGCs stimulation increases alertness level^9^ or slow it down because the neural processing of S-cones is sluggish compared to other photoreceptors^18,19^. Regarding the two hypotheses based on high-level cognitive processing, the positive concepts of blue color (such as openness and tranquility) could aid the concept-related attention shift^26^. In contrast, the connection to the blue light hazard could slow it down. Although these factors have shown their potential to influence cognitive performances, it remains elusive how they affect the effect of blue light on visual-spatial attention shift, which is the primary goal we intend to test here. Furthermore, because the factors mentioned above can potentially affect the processes of both exogenous and endogenous attention, we had no specific hypothesis on how blue light affects the shift speeds of each type of visual attention.

**Table 1:** Four hypotheses and their possible contributions to the effect of blue light on attention shift.

	Low-level perceptual processing	High-level cognitive processing
Facilitatory effect	**ipRGCs**	**Positive associations with the color blue**
	The stimulation of ipRGCs may speed up visual-spatial attention by increasing alertness	Blue is associated with positive concepts (i.e., openness and calmness) and may facilitate the concept-related attention shift
Inhibitory effect	**S-cone**	**Negative associations with the blue light hazard**
	S-cones’ neural processing is delayed relative to other photoreceptors, which may cause visual-spatial attention shift to slow down	The association of the blue light hazard may activate participants’ motivation to hold back and impair their attention shift under blue light

We adopted the refined Wundt’s clock paradigm to estimate how blue light affects the speed of exogenous and endogenous attention shift^38^ because it can directly measure the time cost of attention shift with higher temporal resolution compared to the other two paradigms^38,49,50^. The Wundt’s clock paradigm consists of three conditions: (1) baseline, (2) exogenous cue, and (3) endogenous cue; each condition estimates task performance (1) without attention shift, (2) with exogenous attention shift, and (3) with endogenous attention shift, respectively. We first examine whether blue light facilitates or impairs the speed of attention shift by presenting different background colors (blue vs. green) in Experiment 1 and making blue and green background light iso-luminant for individual participants in Experiment 2. We then use the silent substitution method^51^ to test whether each blue-light sensitive photoreceptor (S-cone and ipRGCs) contributes to the speed of attention shift in Experiment 3 and Experiment 4, respectively.

## Experiment 1

To test the four hypotheses (Table 1), we first investigate whether blue-light exposure accelerates or retards attention shift. If blue light speeds up attention shift, it might be due to ipRGCs stimulation or positive associations with the color blue; if it slows it down, it could be due to S-cone stimulation or an association with a blue light hazard.

### Methods

#### Participants

We recruited 26 male participants (20–35 years old). Females were not included to avoid the possible interaction between the menstrual cycle and the influence of light exposure^52^. Data of participants whose average latency deviated from three standard deviations of the overall average latency for each light and cue condition were excluded. One participant was excluded as an outlier, and one participant dropped out during the experiment, making the final sample size 24. All participants had normal or correct-to-normal vision and did not wear glasses with any blue light filter. They gave informed consent before their participation and were naïve to the purpose of the experiment. All experiments in this study (Experiment 1 ~ Experiment 4) were approved by the Research Ethics Committee of National Taiwan University (NTU-REC 2015HS071) and all methods were performed following applicable research subject guidelines and regulations.

#### Apparatus and stimuli

All stimuli were displayed on an LCD monitor with a 60 Hz refresh rate controlled by a PC running Matlab (The MathWorks, Inc.), using PsychToolbox extensions^53^. The experiment was carried out in a room with no lighting other than that provided by the computer screen. The participant sat with his head on the chinrest at a distance of 57 cm from the monitor to reduce head movement during the experiment.

**Figure 1:**
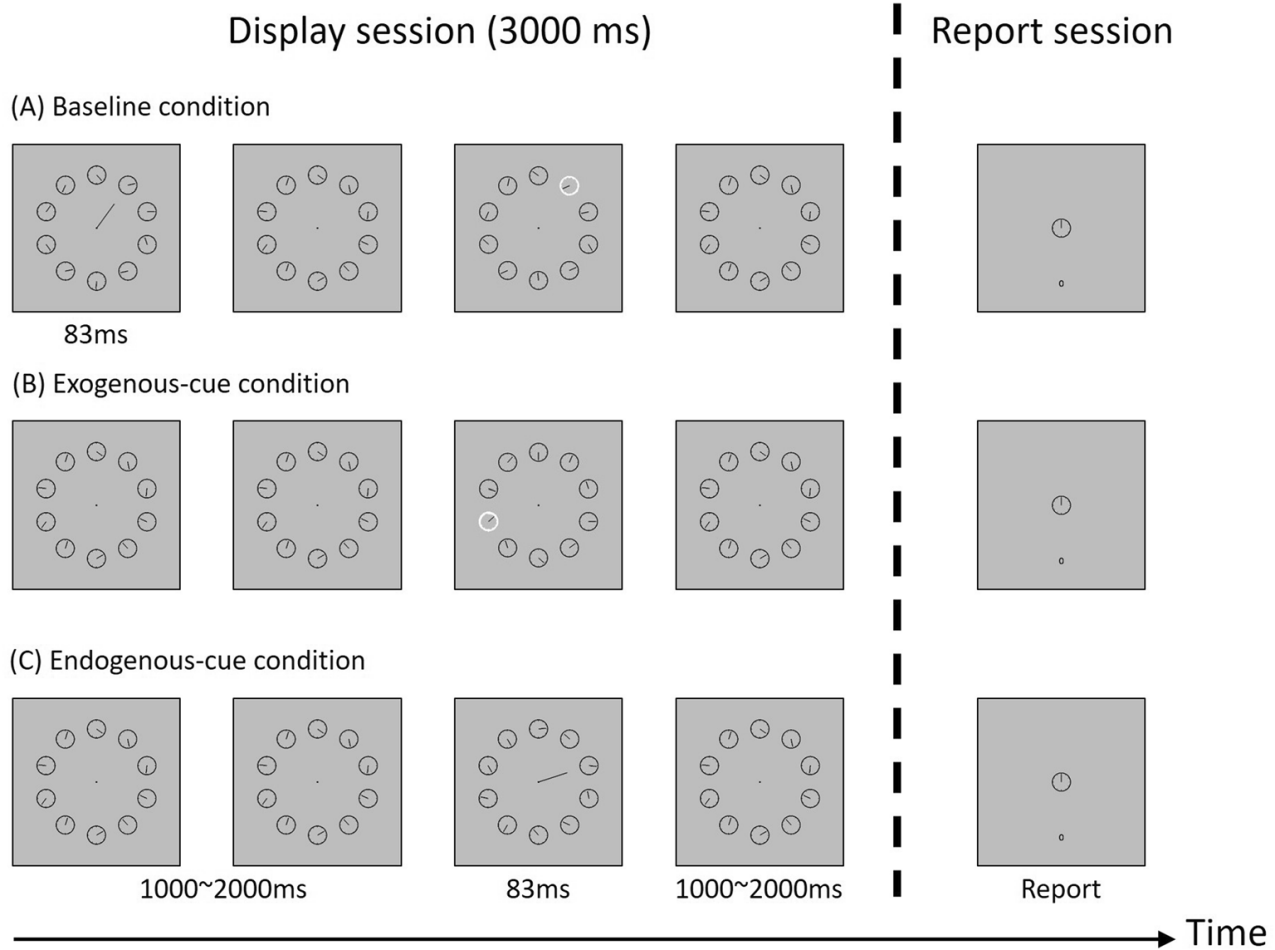
The procedure of the clock paradigm used in this study.

The clock paradigm requires participants to report their answers after the stimuli display (Fig. 1). In the display session, 10 clocks, each with a clockwise-running hand (outlined in black) were displayed surrounding the fixation point in an imagery circle with a diameter of 7°. Each clock was 2.5° of visual angle in diameter and featured a clock hand moving one lap per second. The initial clock time on each clock was randomly assigned in each trial. The clock's rim changed from black to white as an exogenous cue, whereas a 4°-long black cueing line extending from the fixation point to one of the clocks served as an endogenous cue.

In the report session, a clock identical to that shown in the display session was presented at the center of the screen with a number (ranging from 0 to 60) presented below as the clock time to indicate the orientation of the clock hand. Participants were asked to use the arrow keys to increase or decrease the clock time to report as accurately as possible the orientation of the clock hand shown at the time of the cue display. The initial clock time was set to be 0 in each trial, and the four arrow keys, left, right, up, and down, could be used to decrease one unit, increase one unit, decrease five units, and increase five units of the clock time, respectively.

**Figure 2:**
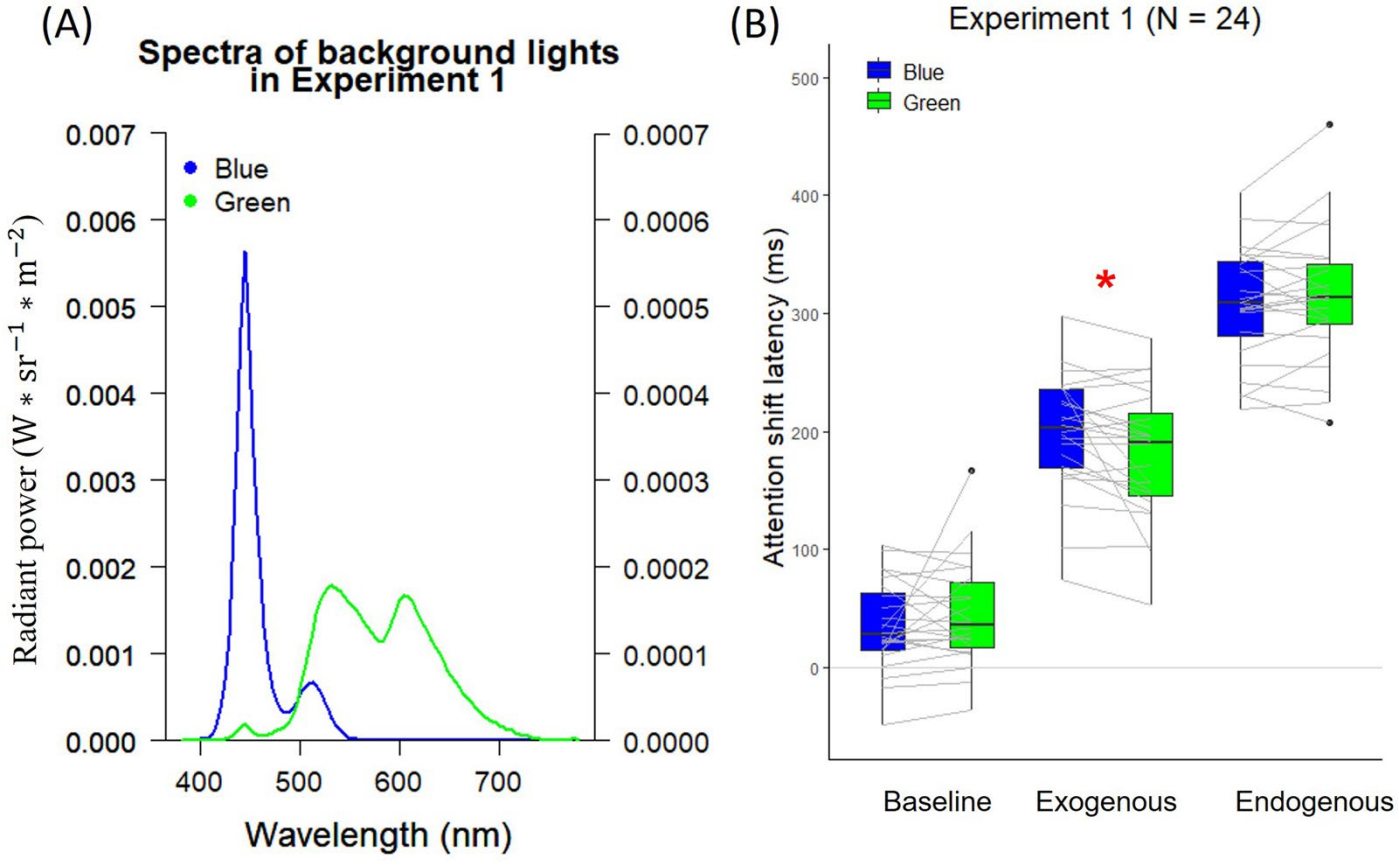
(**A**) Background light spectra used in Experiment 1. The spectra of blue and green background lights are plotted as a function of wavelength (x-axis) in radiant power (y-axis). The radiant power of the blue background light was represented on the left y-axis, while the radiant power of the green background light was represented on the right y-axis. (**B**) Attention shift latencies in different cue conditions under blue and green background lights in Experiment 1. The two ends of grey lines connecting blue and green boxplots indicate the average latencies of each participant under blue and green lights. The post hoc test indicated that the average latencies of the exogenous-attention shift under blue light were slower than under green light. The symbol* indicates that the latencies under blue and green lights differ significantly (Holm adjusted *p* < .05).

Two background lights—blue (test) and green (control)—were employed, and the composition of the two lights was estimated using a PR655 Spectroradiometer (see Fig. 2A for the spectra). The luminance values for the blue and green lights were 21.05 cd/ m^2^ (CIE xy: 0.15, 0.09) and 10.31 cd/ m^2^ (CIE xy: 0.45, 0.50), respectively. Table 2 summarizes the levels of cone and ipRGCs stimulations.

**Table 2:** Stimulation of Cones and ipRGCs in the Four Experiments in this study.

Experiment	Light	L	M	S	ipRGCs
Experiment 1	blue	11.99	9.06	183.19	83.70
	green	7.33	2.97	1.01	5.41
	ratio	1.63	3.05	181.12	15.47
Experiment 2	blue	13.35	10.33	174.92	100.59
	green	10.20	5.37	2.32	13.30
	ratio	1.31	1.92	75.51	7.56
Experiment 3	S-cone high	128.50	38.00	96.37	51.50
	S-cone low	133.49	40.12	27.13	44.38
	ratio	0.96	0.95	3.55	1.16
Experiment 4	ipRGCs high	137.33	42.93	59.51	69.88
	ipRGCs low	126.72	36.98	61.85	30.38
	ratio	1.08	1.16	0.96	2.30

#### Procedure and design

To reduce the influence of the circadian rhythm, participants were requested to participate in the experiment at the same period of time each day on three different days. The procedure and the stimuli were identical across the three days except for the background lights. Participants practiced the task under a grey light on the first day of the experiment to diminish the practice effect in the formal trials. Then, on the second and third days, participants experimented with blue and green lights, with the order being counterbalanced between them.

All experiments started with a 20-min light adaptation. Afterward, participants completed the practice phase with 30 trials, 10 for each cue condition. They then conducted 50 trials in each of the three conditions in the experiment phase. The trials in each condition were mixed and presented in a pseudo-random order. The experiment lasted about 40–60 min.

The procedure of the clock paradigm is illustrated in Fig. 1. The participants were instructed to shift their attention to the cued clock and report as accurately as possible the clock time perceived at the cue onset. In each trial, participants were asked to start the trial by pressing the space key and to remain fixated on the central point during the stimuli display. After each trial began, the clocks were presented for 3000 ms, with either an exogenous or endogenous cue presented for 83 ms at the middle third of the display time. The exogenous cue was used in the baseline condition and the exogenous cue condition, whereas the endogenous cue was used in the endogenous cue condition.

In the baseline condition, different from the other two conditions, participants were informed which clock was the target clock at the beginning of trials by an endogenous cue. This way, we could measure participants’ performance without the time cost of attention shift because spatial attention had been deployed on the target clock before the exogenous cue was presented. Finally, after the stimuli display, participants were asked to report the clock time perceived at the exogenous cue display by adjusting the orientation of the clock hand on it using the four designated keys. The speed of attention shift was estimated by the time latencies between the physical (presented) and perceptual (reported) cue onset time.

### Results

We excluded the trials with estimated attention shift latencies that deviated from three standard deviations of the average latencies under each cue condition (1.29% of trials were excluded). The remaining trials were averaged and illustrated in Fig. 2B. The estimated latencies of the remaining trials were analyzed using a two-way repeated-measures Analysis of Variance (ANOVA) with the within-subject factors of Cue Type (baseline, exogenous, and endogenous) and Light (blue, green).

The ANOVA revealed a main effect of Cue Type, *F*(2, 46) = 424.60, *p* < 0.001, ${\upeta }_{\mathrm{p}}^{2}$ = 0.95. The main effect of Light was not significant, *F*(1, 23) = 0.06, *p* = 0.808, ${\upeta }_{\mathrm{p}}^{2}$ < 0.01. Critically, the interaction between Cue Type and Light was significant, *F*(2, 46) = 4.75, *p* = 0.013, ${\upeta }_{\mathrm{p}}^{2}$ = 0.17. The following post hoc test for the effect of lights of each cue type indicated that the average latencies of the exogenous-cue condition under blue light were significantly slower than those under green light, paired *t*(23) = 2.72, Holm adjusted *p* = 0.036. There was no significant difference observed in the baseline and endogenous-cue conditions, all Holm adjusted *ps* > 0.05.

### Discussion

Experiment 1 replicated the findings of Carlson et al. that the estimated latencies varied depending on the cue conditions^38^. Although the latencies estimated in our study were higher than those in Carlson et al.^38^, they were within the range of values reported in other studies^38–40^. Our findings demonstrated that, compared to green light, blue light slowed down the speed of exogenous attention shift but had no effect on the speed of endogenous attention shift, suggesting that S-cones stimulation and the association with a blue light hazard could be the causes.

## Experiment 2

In Experiment 2, we used heterochromatic flicker photometry (HFP)^54–56^ to minimize individual differences in luminance perception between blue and green lights^57^, which could have influenced our results because luminance contrast affects perceived blur and speed^58,59^ on the moving clock hands. We also used the Karolinska Sleepiness Scale questionnaire (KSS)^60^ to measure participants’ alertness level to see if it was correlated (positively or negatively) with task performance, as shown in previous blue light studies^9,61,62^. Further, we used a linear mixed-effects model (LME) analysis to see how the stimulation of each photoreceptor affected the combined task performances of Experiments 1 and 2 and to provide parameters for predicting likely results in Experiments 3 and 4.

### Methods

#### Participants

Twenty-seven male participants (age range: 20–34 years old) were recruited for Experiment 2. Three participants were excluded from further analysis: (1) One participant wore blue-light-blocking glasses on the third day of the experiment. (2) One participant was exposed to lighting that was out of our experimental planning during the experiment. (3) One participant did not follow the instructions. The final sample size for Experiment 2 was 24 participants. All criteria were the same as in Experiment 1.

#### Apparatus and stimuli

**Figure 3:**
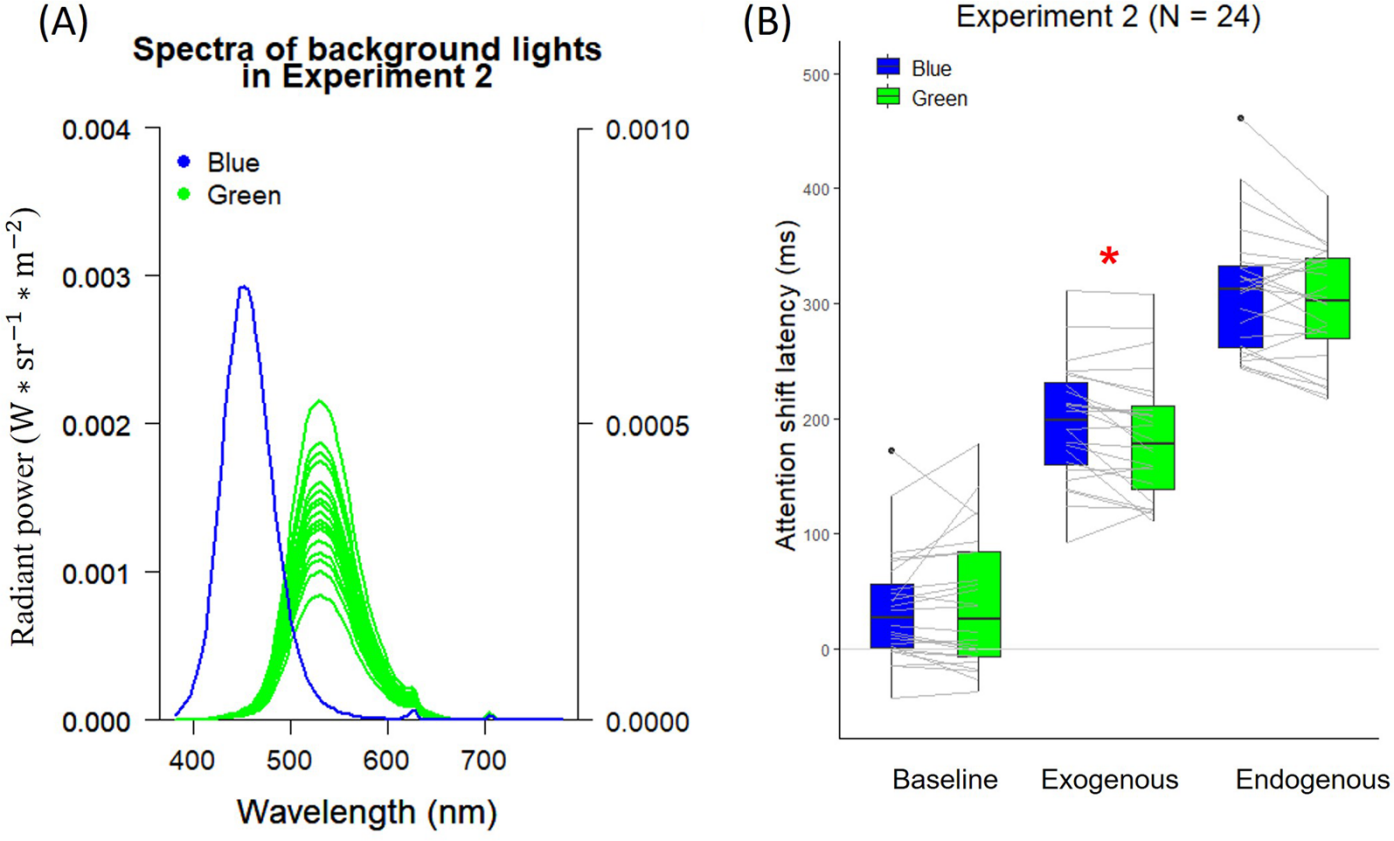
(**A**) Background light spectra used in Experiment 2. The spectrum of blue light and those of individually HFP-matched green background lights are plotted as a function of wavelength (x-axis) in radiant power (y-axis). The radiant power of the blue background light was represented on the left y-axis, while the radiant power of the green background light was represented on the right y-axis. (**B**) Attention shift latencies in different cue conditions under blue and green background lights in Experiment 2. The two ends of grey lines connecting blue and green boxplots indicate the average latencies of each participant under blue and green lights. The post hoc test indicated that the average latencies of the exogenous-attention shift under blue light were slower than under green light. The symbol* indicates that the latencies under blue and green lights differ significantly (Holm adjusted *p* < .05).

All the settings were the same as in Experiment 1 except for the following. First, the stimuli were displayed on a CRT monitor instead of an LCD monitor. Second, the background lights were customized by the HFP to minimize the individual differences in luminance perception^57^. The intensity of blue light used in this experiment was kept constant across the participants, and the intensities of the green light were customized by HFP for each individual to match the luminance of the blue light (23.68 cd/ m^2^). Compositions of blue (CIE xy: 0.14, 0.10) and green (CIE xy: 0.32, 0.59) lights can be seen in Fig. 3A for the spectra. Table 2 summarizes the levels of cone and ipRGCs stimulation.

#### Procedure and design

As previously stated, individuals were requested to participate in the experiment on three different days. Participants conducted the HFP procedure to establish their equal-luminance point for green and blue lights on the first day and then practiced the clock paradigm in the grey light.

In the HFP procedure, a filled circle (2° in diameter) presented at the center of the screen alternatively changed its color between blue and green with a frequency of 15 Hz on a black background to silence the chromatic change discriminability. In this way, participants could perceive only luminance change between two lights in such settings. The intensity of the blue light was kept constant, and that of the green light was randomly assigned to an initial level and adjustable. Participants were introduced to adjust the intensity of the green light to make the two lights fused to a stable image as much as possible. Once the two lights fused, the perceived luminance of the green light would be very close to that of the blue light as adjusted individually. The participants completed the HFP five times, and the median intensity of the matched green lights was used in the experiment.

On the second and third days, participants performed the tasks under the HFP-matched blue and green lights. The order of the two lights was counterbalanced between participants. Participants were instructed to indicate their subjective alertness level before and after the clock paradigm by selecting one of the options in a KSS questionnaire that best described their alertness level at the time. The options and descriptions ranged from 1 (extremely alert) to 9 (very sleepy, great effort to keep alert, fighting sleep). The rest of the procedure was the same as in Experiment 1.

Furthermore, the LME analysis was carried out to investigate how photoreceptors, especially S-cone and ipRGCs, contributed to the task performances and to provide parameters for predicting likely results in the following experiments. This was done by using the ‘lme4’^63^ and ‘lmerTest’^64^ packages from the statistical analysis software R^65^. The data from Experiments 1 and 2 were combined in the analysis. The analysis comprised three steps: (1) Compare the full and null models using the likelihood ratio test to check whether the stimulation of the photoreceptors could predict the task performances in each cue condition. (2) Systematically reduce the fixed effects of the full model to avoid over-specification in the LME models. (3) Calculate the slope of the determined models to generate parameters for the subsequent experiments' prediction. The formula for both the full and null models are:

1 $${\text{Full model}}:{\text{latency}}\sim {\text{L}} + {\text{M}} + {\text{S}} + {\text{ipRGCs}} + \left( {{1}|{\text{subject}}} \right)$$

2 $${\text{Null model}}:{\text{latency}}\sim \left( {{1}|{\text{subject}}} \right)$$

where L, M, S, ipRGCs are the amounts of stimulation for L-cones, M-cones, S-cones, and ipRGCs.

### Results

We excluded the trials where the estimated attention shift latencies deviated from three standard deviations of the average latencies under each cue condition (1.03% of trials were removed). The remaining trials were averaged and illustrated in Fig. 3B. The estimated latencies of the remaining trials were analyzed using a two-way repeated-measures ANOVA with factors of Cue Type (baseline, exogenous, endogenous) and Light (blue, green).

Similar to the results reported in Experiment 1, ANOVA revealed a significant main effect of Cue Type, *F*(2, 46) = 456.10, *p* < 0.001, ${\upeta }_{\mathrm{p}}^{2}$ = 0.95. The main effect of Light was not significant, *F*(1, 23) = 3.98, *p* = 0.058, ${\eta }_{p}^{2}$ = 0.15. Critically, the interaction between Cue Type and Light was significant, *F*(2, 46) = 3.96, *p* = 0.026, ${\upeta }_{\mathrm{p}}^{2}$ = 0.15. The following Post hoc test for the effect of lights of each cue type indicated that the average latencies in the exogenous-cue condition under blue light were significantly higher than those under green light, paired *t*(23) = 2.84, Holm adjusted *p* = 0.028. There was no significant difference in the baseline and endogenous-cue conditions, all Holm adjusted *ps* > 0.05.

**Figure 4:**
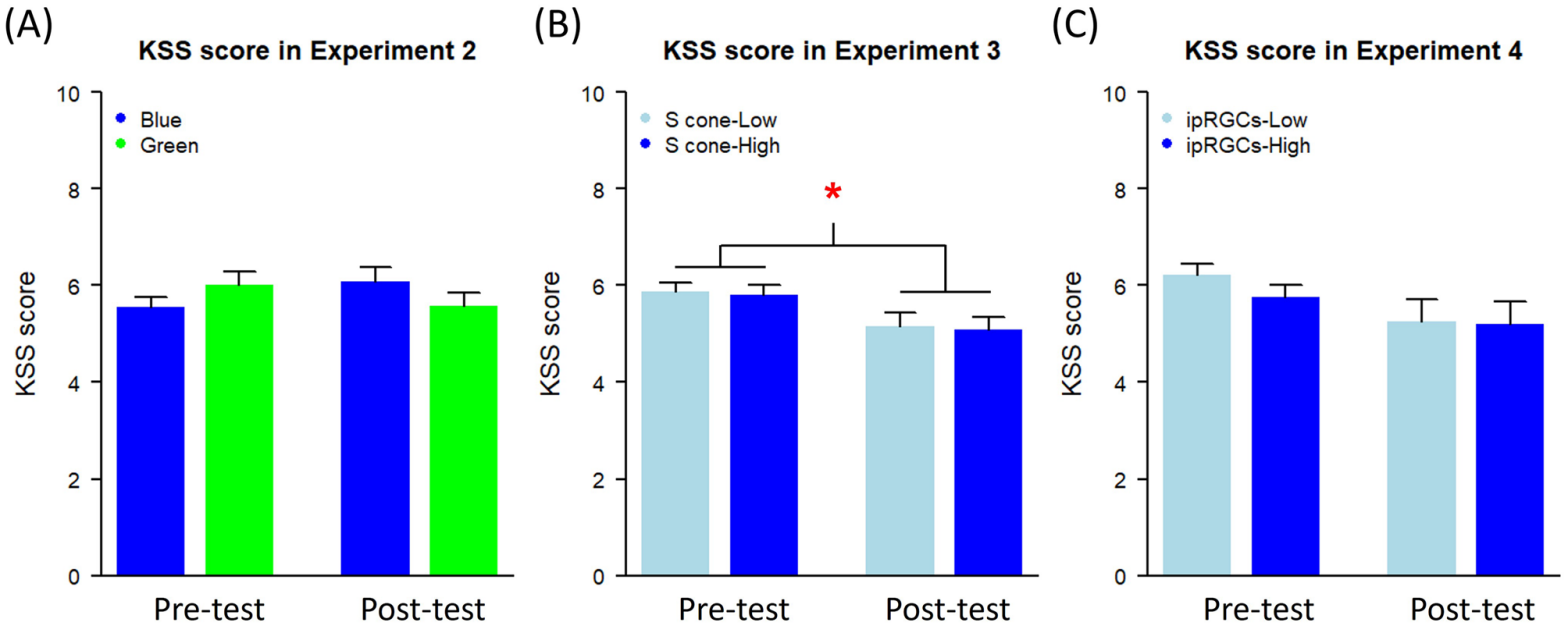
KSS scores in different time points under different background lights in (**A**) Experiment 2, (**B**) Experiment 3, and (**C**) Experiment 4. Error bars depict standard errors of the mean. The symbol * indicates a significant difference in KSS scores between different time points in Experiment 3 (*p* < .05).

A two-way repeated-measures ANOVA on the KSS scores with the factors of Light (blue, green) and Time (pre-task, post-task) revealed that there was neither interaction nor main effects of Light and Time, all *ps* > 0.05 (see Fig. 4A). We also used linear regression analysis to investigate the relations between the performance differences between blue and green background lights on the attention shift latency of each cue type and the KSS scores before and after the task. The results showed that the linear regression models of all combinations were not significant (all *ps* > 0.05).

The results of LME for the combined data (Experiments 1 and 2) revealed that the stimulation of photoreceptors could only predict the performance in the exogenous-cue condition,${\chi }^{2}$(4, N = 48) = 13.96, *p* = 0.007, but not in the baseline condition, ${\chi }^{2}$(4, N = 48) = 2.45, *p* = 0.654, nor the endogenous-cue condition,${\chi }^{2}$(4, N = 48) = 8.63, *p* = 0.071. After reducing other fixed effects, two models, (1) S-cone model (model: latency ~ S + (1|subject)) and surprisingly (2) ipRGCs model (model: latency ~ ipRGCs + (1|subject)), were determined and tested in the following steps. The goodness of fit of both models were significantly higher than the null model (S-cone model: ${\chi }^{2}$(1, N = 48) = 13.42, *p* < 0.001; ipRGCs model:${\chi }^{2}$(1, N = 48) = 13.03, *p* < 0.001), and similar with the full model (S-cone model: ${\chi }^{2}$(3, N = 48) = 0.55, *p* = 0.909; ipRGCs model:${\chi }^{2}$(3, N = 48) = 0.93, *p* = 0.818). The slope of S-cone and ipRGCs models were 0.09 and 0.19, indicating that the shift latencies of exogenous visual attention would slow down 0.09 and 0.19 ms per unit of S-cone and ipRGCs stimulation, respectively.

### Discussion

Experiment 2 used an iso-luminance lighting condition to replicate the results of Experiment 1, revealing that the blue light impairment effect of exogenous attention shift was not attributable to a luminance difference between two background lights. This is consistent with previous findings that luminance affects the perceived speed and blur of moving objects only when the luminance contrast of one moving object is 250 times higher than the other^59^, which is not the case in our study. Notably, the impairment we found here was independent of the participants’ alertness level. The LME analysis revealed that the stimulations of S-cones and ipRGCs could be used to predict the blue light impairment of the speed of exogenous attention shift. As a result, we isolated these factors in the following experiments to see what factor(s) contribute to the blue-light effect on the speed of exogenous attention shift.

## Experiment 3

In Experiment 3, we investigate how the S-cone stimulation of background lights influenced the speed of attention shift. The S-cone could contribute to the blue-light impairment we found due to the sluggish property of its processing^18,19^ and its poor ability to induce exogenous and endogenous attention shifts^66,67^, compared with other photoreceptors. We used a multi-primary system that can separately control the stimulations of three types of cones and ipRGCs by using color additive mixing methods ^68–70^ (see^11^ for the structure of the multi-primary system). We were able to manipulate the stimulation of S-cones while keeping the background color and stimulation of other photoreceptors constant by using this system.

### Methods

#### Participants

We initially recruited 31 male participants (ages 20–33); however, one participant was eliminated as an outlier, and six others dropped out during the trial, resulting in a final sample size of 24 participants. Criteria for recruitment were the same as in Experiment 1.

#### Apparatus and stimuli

All stimuli were displayed on the multi-primary system with a 60 Hz refresh rate. The participant sat at a distance of 30 cm from the display. All the stimuli in the clock paradigm were identical to those in Experiment 2 except for the background lights.

**Figure 5:**
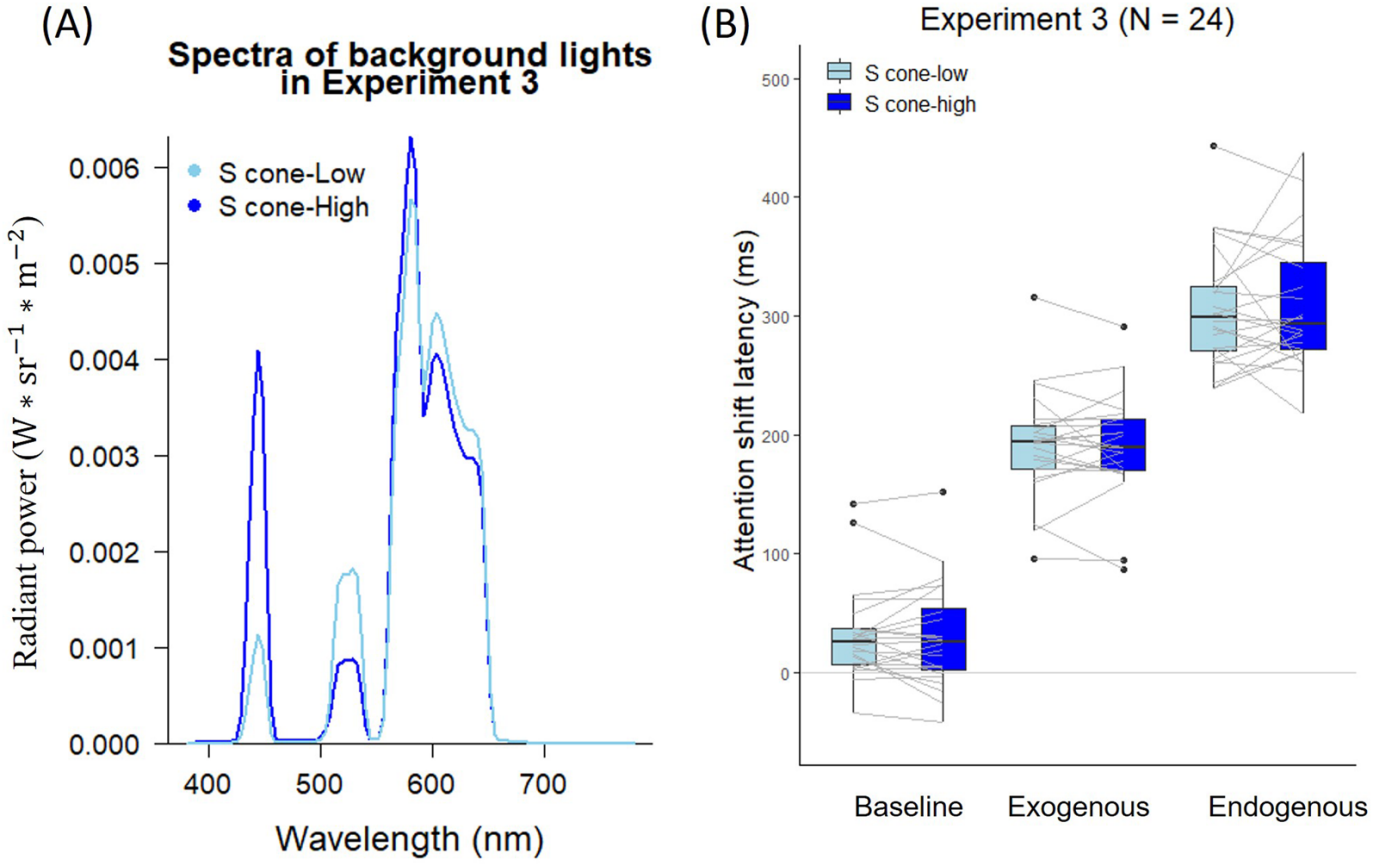
(**A**) Background light spectra used in Experiment 3. The spectra of low and high S-cone stimulating background lights are plotted as a function of wavelength (x-axis) in radiant power (y-axis). (**B**) Attention shift latencies in different cue conditions under low and high S-cone stimulating background lights in Experiment 3. The two ends of grey lines connecting two boxplots indicate the average latencies of each participant under the low and high S-cone stimulating lights.

For the multi-primary system, the lights of the three projectors passed through the corresponding interference filters, creating four primaries of lights (peak wavelengths: 455 nm, 530 nm, 580 nm, and 595 nm), and then overlapped and projected to the screen in front of the participant. We adjusted the light intensities of the projectors to create planned background lights using color additive mixing methods. To manipulate the stimulation of S-cones, two lights corresponding to two conditions, S-cone-low (luminance: 173.61 cd/m^2^; CIE xy: 0.53, 0.41) and S-cone-high (luminance: 166.49 cd/m^2^; CIE xy: 0.47, 0.34), were created by using the multi-primary system (see Fig. 5A for the spectra). The stimulation of S-cones between these two conditions was manipulated while keeping the stimulation of other photoreceptors constant (see Table 2 for details).

#### Procedure and design

Participants came to the lab on three different days. Participants practiced the task on an LCD screen with a grey light background on the first day. The formal tasks for S-cone-low and S-cone-high conditions were completed on the second and third days. The order of the S-cone-low and S-cone-high conditions was counterbalanced between participants. The rest of the procedure followed the same pattern as the second and third days of Experiment 2.

With the background lights of Experiment 3, we calculated the expected S-cone effect on the speed of exogenous attention shift. We compared it to the observed S-cone effect to evaluate if the impairment effect of blue light on exogenous attention shift was induced by S-cone stimulation. The expected S-cone effect was determined using the parameter calculated from the LME analysis in Experiment 2, which predicted that exogenous visual attention shift latencies would slow by 0.09 ms per unit of S-cone stimulation. The mechanism of the impairment effect of blue light would be distinct from that of the S-cone effect if the observed and expected S-cone effects are different; if not, they could be the same process.

### Results

The trials in which the estimated attention shift latencies differed by more than three standard deviations from the average latencies under each condition were excluded (1.33% of trials were removed). Figure 5B shows the averaged results of the remaining trials. The estimated latencies of the remaining trials were analyzed using a two-way repeated-measures ANOVA with the factors of Cue Type (baseline, exogenous, and endogenous) and Light (S-cone-low, S-cone-high). Same as previous experiments, the main effect of Cue Type was found, *F*(2, 46) = 351.80, *p* < 0.001,${\upeta }_{\mathrm{p}}^{2}$ = 0.94. However, there was neither the interaction between Cue Type and Light, *F*(2, 46) = 0.26, *p* = 0.776, ${\upeta }_{\mathrm{p}}^{2}$ = 0.011, nor the main effect of Light, *F*(1, 23) = 0.02, *p* = 0.879, ${\upeta }_{\mathrm{p}}^{2}$ < 0.01.

The result of the one-sample *t*-test revealed that the observed S-cone effect on the speed of exogenous attention shift (*M* = −0.80 ms, *SD* = 23.39) did not significantly differ from the expected value (6.34 ms) calculated by the slope from the S-cone model in Experiment 2, *t*(23) = −1.49, *p* = 0.149.

A two-way repeated-measures ANOVA on the KSS scores with the factors of Light (S-cone-low, S-cone-high) and Time (pre-task, post-task) revealed the main effect of Time, *F*(1, 23) = 9.73, *p* = 0.005, ${\upeta }_{\mathrm{p}}^{2}$ = 0.30, which showed that participants’ alertness level increased during the task. However, there was neither the interaction between Light and Time nor the main effect of Light, all *ps* > 0.05 (see Fig. 4B). These findings are consistent with those of Spitschan et al., who found that S-cone stimulation had no effect on both subjective and objective alertness^71^.

### Discussion

Experiment 3 showed that manipulating S-cone stimulation did not affect the speed of both exogenous and endogenous attention shifts when we eliminated the color difference of background lights. The observed S-cone effect on exogenous attention shift, however, did not deviate from the expected value calculated from Experiments 1 and 2. Based on these, S-cone stimulation does not contribute to the impairment of exogenous attention shift we found in Experiments 1 and 2.

The multi-primary system's restriction (3.55 times of S-cone manipulation between metameric lights) may have prevented the manipulation of S-cone stimulation from reaching a certain threshold, which may provide an alternative explanation as to why the S-cone effect was not observed here. This is in contrast to what is possible in Experiments 1 and 2 with perceived color differences (181.12 and 75.51 times of S-cone manipulation between blue and green lights in Experiments 1 and 2, respectively). That is, higher S-cone stimulation than what can be done with the metameric lights by our multi-primary system here means that one of the background colors would be perceived as more bluish than the other, which might defeat our purpose of examing the effect of S-cone stimulation at the receptor level activation without perceived color differences. Future research is needed to see whether larger manipulations of S-cone stimulations could impair the speed of exogenous visual attention shifts and how its color modulates this effect.

## Experiment 4

Experiment 4 examines whether ipRGCs stimulation of background lights slowed the speed of attention shift as predicted by the ipRGCs model established in Experiment 2. It is also possible that the ipRGCs worked, but their effect was canceled by other factors such as the S-cone stimulation in blue lights. With the color-difference-eliminated metameric background lights, we would revisit the hypothesis that ipRGCs stimulation could speed up visual-spatial attention by increasing alertness. Using the multi-primary system, we were able to manipulate the stimulation of ipRGCs while keeping the stimulation of other photoreceptors at an identical level.

### Methods

#### Participants

We recruited a total of 27 male participants (age range: 20–34 years old); however, one participant’s data was excluded as an outlier, and two participants dropped out during the experiment. The final sample size for Experiment 4 was 24. All criteria were the same as in Experiment 1.

#### Apparatus and stimuli

The apparatus and stimuli used in Experiment 4 were identical to those in Experiment 3 except for the background lights.

**Figure 6:**
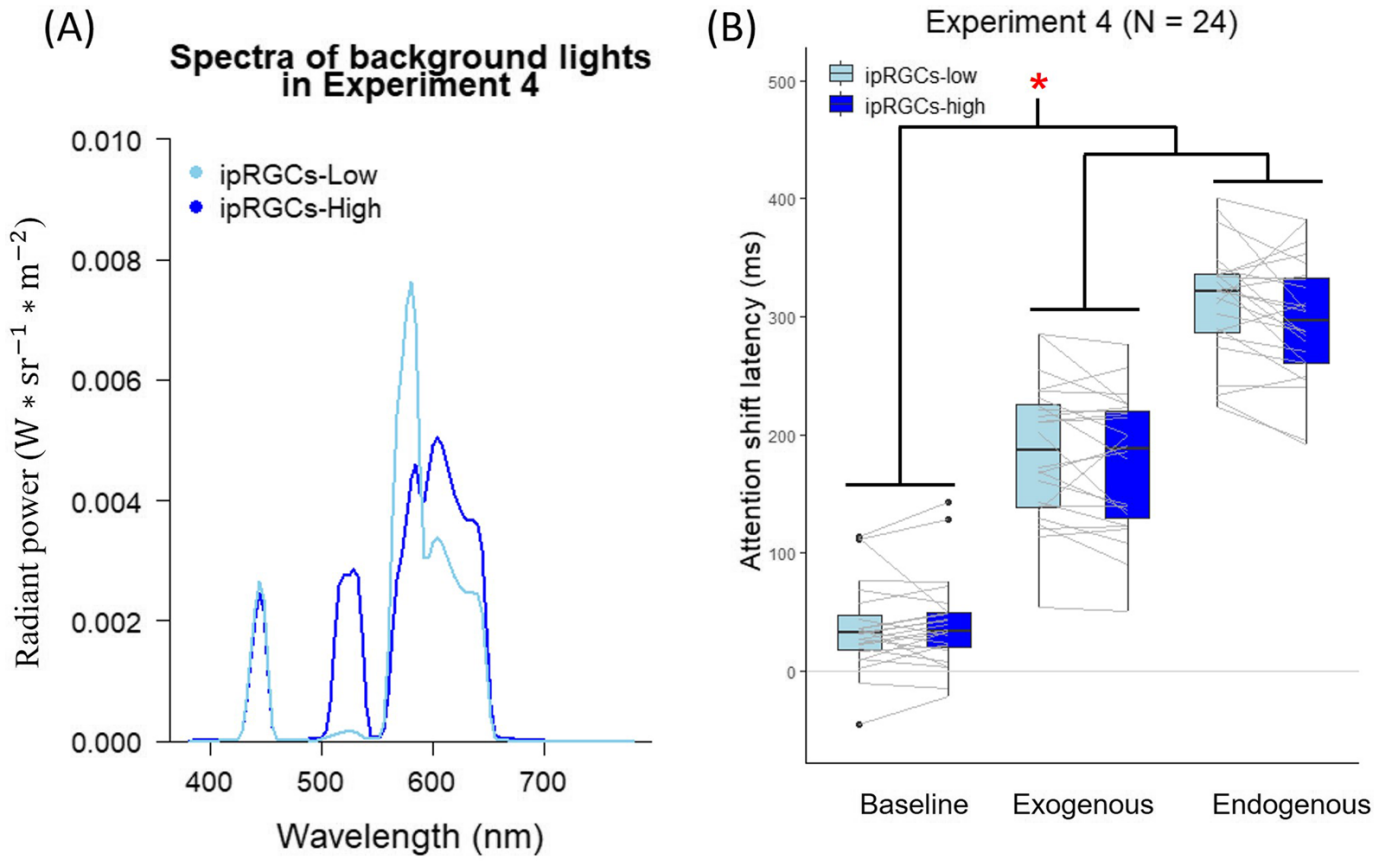
(**A**) Background light spectra used in Experiment 4. The spectra of low and high ipRGCs stimulating background lights are plotted as a function of wavelength (x-axis) in radiant power (y-axis). (**B**) Attention shift latencies in different cue conditions under low and high ipRGCs stimulating background lights in Experiment 4. The two ends of grey lines connecting two boxplots indicate the average latencies of each participant under the low and high ipRGCs stimulating lights. The contrast-contrast interaction indicated that the ipRGCs stimulation accelerated both the exogenous and endogenous attention shifts. The symbol* indicates that the ipRGCs effect between two groups of conditions, one is the baseline condition, and the other is the exogenous-cue and endogenous-cue conditions, differ significantly (*p* < .05).

To manipulate the stimulation of ipRGCs, two background lights corresponding to two conditions, ipRGCs-low (luminance: 163.70 cd/m^2^; CIE xy: 0.50, 0.36) and ipRGCs-high (luminance: 180.26 cd/m^2^; CIE xy: 0.49, 0.38), were created by using the multi-primary system (see Fig. 6A for the spectra). The stimulation of ipRGCs between these two conditions was manipulated while keeping the stimulation of other photoreceptors constant (see Table 2 for details).

#### Procedure and design

The procedure and design were the same as in Experiment 3.

To see if the effect of ipRGCs stimulation and the impairing effect of blue light were caused by the same mechanism, we calculated the expected and observed ipRGCs effect on the speed of exogenous attention shift. The parameter calculated from the LME analysis that the shift latencies of exogenous visual attention would slow down 0.19 ms per unit of ipRGCs stimulation was used as the coefficient for estimating the expected ipRGCs effect.

### Results

We excluded the trials where the estimated latencies deviated from three standard deviations of the average latencies under each condition (1.36% of trials were removed). The remaining trials were averaged and illustrated in Fig. 6B. The estimated latencies of the remaining trials were analyzed using a two-way repeated-measures ANOVA with Cue Type (baseline, exogenous, and endogenous) and Light (ipRGCs-low, ipRGCs-high). The main effect of Cue type was significant, *F*(2, 46) = 598.50, *p* < 0.001,${\upeta }_{\mathrm{p}}^{2}$ = 0.96. The main effect of Light was not significant, *F*(1, 23) = 3.62, *p* = 0.070,${\upeta }_{\mathrm{p}}^{2}$ = 0.14. Critically, the interaction between Cue type and Light was significant, *F*(2, 46) = 3.58, *p* = 0.036,${\upeta }_{\mathrm{p}}^{2}$ = 0.13. However, the post-hoc test for the effect of background lights in each cue type showed that there was no significant difference between the ipRGCs-low and the ipRGCs-high conditions in all the cue conditions (baseline: paired *t*(23) = −0.54, Holm adjusted *p* = 1; exogenous: paired *t*(23) = 1.67, Holm adjusted *p* = 0.327; endogenous: paired *t*(23) = 2.30, Holm adjusted *p* = 0.094).

Although the manipulation of ipRGCs stimulation did not significantly affect the performance in every single cue condition, the interaction effect could be attributed to the contrast-contrast interaction (also called interaction contrast)^72^. The interactions between the contrasts of the two factors of interest were evaluated for the contrast-contrast interaction to provide more advanced information than the simple post-hoc test. We therefore tested the contrast-contrast interaction for the two factors: Light and Cue Type. On the one hand, one contrast was tested for the Light, ${\widehat{\mathrm{\varphi }}}_{\mathrm{L}}$(ipRGCs low, ipRGCs high): ${\widehat{\mathrm{\varphi }}}_{\mathrm{L}}$(−1,1). The ${\widehat{\mathrm{\varphi }}}_{\mathrm{L}}$ represented the ipRGCs levels we defined above. On the other hand, according to our hypotheses, two contrasts were tested for Cue Type, ${\widehat{\mathrm{\varphi }}}_{{\mathrm{h}}_{\mathrm{i}}}$(baseline, exogenous, endogenous): ${\widehat{\mathrm{\varphi }}}_{{\mathrm{h}}_{1}}$(−1,2,−1), and ${\widehat{\mathrm{\varphi }}}_{{\mathrm{h}}_{2}}$(2,−1,−1). The ${\widehat{\mathrm{\varphi }}}_{{\mathrm{h}}_{\mathrm{i}}}$ represents the contrast of two groups of conditions we defined. The ${\widehat{\mathrm{\varphi }}}_{{\mathrm{h}}_{1}}$ represents the hypothesis that the ipRGCs stimulation of lights slows the speed of exogenous attention shift whereas the ${\widehat{\mathrm{\varphi }}}_{{\mathrm{h}}_{2}}$ represents the hypothesis that the ipRGCs stimulation of lights accelerates the speed of both exogenous and endogenous attention shifts through alertness increment.

The results indicated that (1) the ${\widehat{\mathrm{\varphi }}}_{{\mathrm{h}}_{1}}$ did not interact with ${\widehat{\mathrm{\varphi }}}_{\mathrm{L}}$, *F*(1,46) = 0.19, *p* = 0.664,${\upeta }_{\mathrm{p}}^{2}$ < 0.01, and (2) the ${\widehat{\mathrm{\varphi }}}_{{\mathrm{h}}_{2}}$ significantly interacted with ${\widehat{\mathrm{\varphi }}}_{\mathrm{L}}$, *F*(1,46) = 6.27, *p* = 0.016,${\upeta }_{\mathrm{p}}^{2}$ = 0.12. The effect of ipRGCs manipulation for the exogenous-cue and endogenous-cue conditions was significantly different from those for the baseline condition. That is, the ipRGCs stimulation accelerated the speed of both the exogenous and endogenous attention shifts as compared to the baseline.

The result of the one-sample *t*-test revealed that the observed ipRGCs effect on the speed of exogenous attention shift (*M* = -9.72 ms, *SD* = 28.56) significantly differed from the expected effect (7.48 ms), *t*(23) = -2.95, *p* = 0.007, suggesting that the effect of ipRGCs and the impairing effect of blue light on exogenous attention shift were caused by different mechanisms.

A two-way repeated-measures ANOVA of the KSS scores on the factors of Light (ipRGCs-low, ipRGCs-high) and Time (pre-task, post-task) revealed that there was neither interaction nor main effects of Light and Time, all *ps* > 0.05 (see Fig. 4C). Furthermore, linear regression analysis revealed that the effect of ipRGCs on attention shift latencies was independent of that on alertness level, all *ps* > 0.05.

### Discussion

Experiment 4 demonstrated that light stimulation of ipRGCs accelerated the speed of both exogenous and endogenous visual attention shift, which differed from the expectation estimated from Experiments 1 and 2. This shows that the stimulation of ipRGCs did not contribute to the blue light impairment we observed. The large amounts of inhibitory outputs from S-cones brought on by blue light may neutralize the effects of ipRGCs in Experiments 1 and 2. The manipulation of ipRGCs stimulation kicked in and accelerated the shifts in visual-spatial attention once the inhibitory outputs from S-cones were equalized between metameric lights. Furthermore, the effect of ipRGCs on the visual attention shift was independent of the participants' alertness level, indicating that the facilitation effect of ipRGCs was not attributable to an increase in alertness.

## General discussion

The goal of this research was to see if and how blue light affected visual-spatial attention shifts. We tested exposure to blue and green light backgrounds and found that exposure to blue light slowed the exogenous visual attention shift (Experiments 1 and 2). To further clarify the contributions of blue-light sensitive photoreceptors (i.e., S-cone, ipRGCs), we applied the multi-primary system to isolate the contribution of a single type of photoreceptors (i.e., the silent substitution method). The results showed that when the color difference was eliminated, but the differences in stimulation levels of S-cones (Experiment 3) or ipRGCs (Experiment 4) remained, the blue light impairment effect on exogenous attention shift vanished. Across all experiments, the light manipulation did not affect participants’ alertness levels. We found a novel blue light impairment of exogenous attention shift caused by color rather than alertness, S-cone stimulation, or ipRGCs stimulation.

### Possible contributions from the basic visual processing of blue and green lights

Some may argue that differences in low-level visual processing, such as acuity, chromatic contrast, and luminance contrast between blue and green lights, could contribute to the impairment effect of blue light on the exogenous attention shift we found here; however, this is unlikely. Although the acuity of blue light, compared to green light, is worse at the fovea and better at the periphery, they are similar at 2° to 15° eccentricity^73^. In the present study, the processing of the exogenous cue and the clocks presented at 7° eccentricity would not confound with the acuity difference between blue and green lights. Moreover, it was found that the chromatic and luminance contrasts could influence the speed of visual-spatial attention but only when the luminance contrast is less than 30%^67,74,75^. However, the luminance contrasts between the exogenous cues and the background lights were all far over 30% across our experiments, making our results immune from the influences of chromatic and luminance contrasts.

### The effect of ipRGCs

We found that the ipRGCs stimulation of metameric background lights accelerated both exogenous and endogenous attention shifts. However, the facilitation effect of ipRGCs was independent of participants’ alertness level. Anatomical studies could add to our findings that some brain areas, such as superior colliculus (SC), receive signals directly from ipRGCs without being mediated by alertness-related brain areas^75,76^. Signals, such as ipRGCs stimulation of background lights, could modulate the activities of frontal eye field (FEF) and improve the performances of eye movements and covert attention^77^ via the SC–mediodorsal thalamus–FEF ascending pathway^78,79^. Moreover, functional Magnetic Resonance Imaging (fMRI) studies provide evidence that the ipRGCs stimulation of metameric lights can boost the blood-oxygen-level-dependent (BOLD) responses of FEF^80^. Lee and Yeh also found that blue-light exposure facilitated saccadic eye movements and attentional disengagement^4^. Since they did not directly manipulate the stimulation of ipRGCs, our current study provides empirical evidence that, although eliminated by the impairment effect of blue color, ipRGCs' stimulation of background lights could facilitate cognitive performances related to FEF, such as exogenous and endogenous visual attention shifts^77^.

### The effect of color overwhelms the effect of ipRGCs

Blue-light exposure is a popular method that can stimulate ipRGCs dozens of times greater in magnitude than other colored lights to investigate the effect of ipRGCs^4,44^; however, by definition, it is coupled with the effect of colors. Only a few studies, however, conducted a series of experiments to separate the effects of color and ipRGCs on cognitive performance^3,11,81^. For instance, Yeh and colleagues in two studies observed that, on the one hand, the subjective time expansion caused by blue light was due to the ipRGCs stimulation^11^, and on the other hand, the effects of blue light on multisensory integration are contributed by processing speed differences between blue and red colors^81^. Still, the relation between the effect of color and ipRGCs is unclear. The current study found that blue light delayed the exogenous attention shift and that stimulation of ipRGCs accelerated both exogenous and endogenous attention shifts. It is reasonable that the blue light used in this study also activated the facilitatory effect of ipRGCs but was canceled by the effect of colors. Our finding provides evidence that the contribution of blue color dominates over that of blue-light sensitive photoreceptors on visual attention shift and suggests that the influence of color on cognitive processing should not be overlooked.

### The effect of blue light hazard

Following the advanced development and popularity of electronics, public opinion on blue light is turning negative, particularly in countries where electronic penetration is high, such as Taiwan. Due to news and advice from the media that exposure to blue-enriched light sources such as LEDs may be damaging to the eyes^82–84^, people begin to associate blue light with retinal damage (i.e., blue light hazard) rather than its benefits (i.e., light therapy). Although the meta-analysis found that blue-light-blocking lenses did not improve eye health^82^, people continue to wear blue-light-blocking glasses and use the blue light filter mode on their cellphones and laptops^85^. This fear was demonstrated in a small-sample (n = 20) pre-test survey from our prior research, in which 50% and 80% of the participants, respectively, reported using blue light glasses and turning on the blue light filters of devices. Additionally, it was shown that up to 95% of participants had a negative attitude toward blue light, even if there was no obvious trend for their predictions about whether it helped or hurt their performances. Just 10% of participants failed to connect blue lights to blue light hazards or blue light glasses/filters in a free-association task. The surging negative attitude toward blue light could activate participants’ motivation to hold back and slow down their visual-spatial attention shift. As a result of our findings, several of the previously documented blue-light effects on cognitive performance may need to be reevaluated and reconsidered.

### The selective effect of blue light hazard on exogenous attention shift

Previous studies indicated that exogenous attention is more receptive to unconscious information than endogenous attention^86^. Moreover, it has been shown that the learned association with colors affected cognitive performances unconsciously, which was sometimes contrary to participants’ expectations^26^. The influence of blue light hazard selectively affected the exogenous attention shift reported in the current study could be due to exogenous attention’s sensitivity and susceptibility to unconscious information.

## Conclusion

Our study demonstrates a novel blue-light color impairment effect on exogenous attention shift that is not caused by alertness, S-cone stimulation, and ipRGCs stimulation, indicating that concept-related blue color contributes more to exogenous attention shift than blue-light sensitive photoreceptors. The impact of blue light on cognitive processing, such as the exogenous attention shift observed here, should be investigated further, particularly in this era of greater daily exposure to blue light and the alarmist call of the blue light hazard. Future studies should consider whether applying conditioning learning or framing to strengthen the positive connection of blue light can spare people from the delayed attention shift effect under blue light.

## Data Availability

Data and the code of the Experiments are available from the following link: https://osf.io/6a8cd/?view_only=5e750829db92481b87f4328fde0546de.
